# A team-based approach to warfarin management in long term care: A feasibility study of the MEDeINR electronic decision support system

**DOI:** 10.1186/1471-2318-10-38

**Published:** 2010-06-10

**Authors:** Alexandra Papaioannou, Courtney C Kennedy, Glenda Campbell, Jacqueline B Stroud, Luqi Wang, Lisa Dolovich, Mark A Crowther

**Affiliations:** 1 Department of Medicine, McMaster University, 1280 Main Street West, Hamilton, L8N 3Z5, Canada; 2 Medical Pharmacies Group Inc., 590 Granite Court, Pickering, L1W 3X6, Canada; 3 Thrombosis Research, St. Joseph's Healthcare, 50 Charlton Avenue East, Hamilton, L8N 4A6, Canada; 4 Department of Family Medicine, McMaster University, 1280 Main Street West, Hamilton, L8N 3Z5, Canada

## Abstract

**Background:**

Previous studies in long-term care (LTC) have demonstrated that warfarin management is suboptimal with preventable adverse events often occurring as a result of poor International Normalized Ratio (INR) control. To assist LTC teams with the challenge of maintaining residents on warfarin in the therapeutic range (INR of 2.0 to 3.0), we developed an electronic decision support system that was based on a validated algorithm for warfarin dosing. We evaluated the MEDeINR system in a pre-post implementation design by examining the impact on INR control, testing frequency, and experiences of staff in using the system.

**Methods:**

For this feasibility study, we piloted the MEDeINR system in six LTC homes in Ontario, Canada. All128 residents (without a prosthetic valve) who were taking warfarin were included. Three-months of INR data prior to MEDeINR was collected via a retrospective chart audit, and three-months of INR data after implementation of MEDeINR was captured in the central computer database. The primary outcomes compared in a pre-post design were time in therapeutic range (TTR) and time in sub/supratherapeutic ranges based on all INR measures for every resident on warfarin. Secondary measures included the number of monthly INR tests/resident and survey/focus-group feedback from the LTC teams.

**Results:**

LTC homes in our study had TTR's that were higher than past reports prior to the intervention. Overall, the TTR increased during the MEDeINR phase (65 to 69%), but was only significantly increased for one home (62% to 71%, p < 0.05). The percentage of time in supratherapeutic decreased from 14% to 11%, p = 0.08); there was little change for the subtherapeutic range (21% to 20%, p = 0.66). Overall, the average number of INR tests/30 days decreased from 4.2 to 3.1 (p < 0.0001) per resident after implementation of MEDeINR. Feedback received from LTC clinicians and staff was that the program decreased the work-load, improved confidence in management and decisions, and was generally easy to use.

**Conclusion:**

Although LTC homes in our sample had TTR's that were relatively high prior to the intervention, the MEDeINR program represented a useful tool to promote optimal TTR, decrease INR venipunctures, streamline processes, and increase nurse and physician confidence around warfarin management. We have demonstrated that MEDeINR was a practical, usable clinical information system that can be incorporated into the LTC environment.

## Background

Warfarin therapy is indicated to reduce the risk of arterial and venous thromboembolism [1] and is a common treatment for many elderly patients [2-4]. When warfarin is properly monitored and maintained within a narrowly defined range, it is a safe and highly effective therapy. For most warfarin indications (atrial fibrillation, deep venous thrombosis, and pulmonary embolism) the optimal therapeutic range, is an International Normalized Ratio (INR) of 2.0 - 3.0 [1]. The consequences of poor INR control are serious and include increased risk for death, myocardial infarction, major bleeding, and stroke [5].

The frail elderly residing in long-term care (LTC) have several prescribing challenges [6-8] with additional barriers specific to anticoagulation such as higher risk for recurrent thromboembolism and major bleeding, polypharmacy, and increased risk of falls [4,9]. Indeed, anticoagulants are one of the most common causes of drug-related adverse events in LTC; the majority of events are attributable to poor anticoagulant control leading to excessive INR's [10-12]. In a study of 25 LTC homes [13], 57% of serious or fatal and 24% of minor warfarin-related adverse events were considered preventable; nearly all of the preventable adverse events occurred in the prescribing and monitoring stages of warfarin therapy. Improved anticoagulation care in the elderly has become a priority for several organizations including the Joint Safety Commission [14].

Despite proven benefits and indication for use regardless of age [1], warfarin is commonly under-prescribed in the elderly [2,15,16]. Even when prescribed, many LTC physicians are uncertain about the appropriate intensity of warfarin and in some cases aim for a subtherapeutic target [17,18]. In an audit we conducted in LTC, a large proportion of the time (27.7%) was spent in the INR range of 1.6-1.9 [19]. A recent meta-analysis reported that patients were safer with a ratio slightly above rather than below the therapeutic range when both hemorrhagic and thromboembolic events were considered, and the authors promote the importance of using computer-based algorithms to avoid systemic under-anticoagulation [18,17]. In the community setting, anticoagulation clinics are often used to manage patients on warfarin, however in the LTC setting other options must be explored.

Utilizing a 'knowledge to action' framework [20,21], whereby "the process of translating knowledge to action is an iterative, dynamic and complex process", this project was designed to address 3 objectives: 1) the application of evidence to improve warfarin care in LTC settings 2) develop an electronic decision support system that facilitates the prescribing and monitoring stages of warfarin care in LTC and 3) provide training and support to promote uptake of the tools and overcome implementation barriers. Thus we developed the MEDeINR evidence-based computer system for assisting LTC teams with warfarin management and conducted a feasibility study to examine its implementation in six pilot LTC homes. The primary outcome we compared in a pre-post implementation design was the percentage of time in the therapeutic range (TTR), subtherapeutic range, and supratherapeutic range. Our secondary objectives were to examine whether the number of monthly INR tests per resident changed after implementing the MEDeINR program and to obtain feed-back from LTC staff on using the MEDeINR system.

## Methods

This project involved a partnership between clinician researchers and a large pharmacy provider (Medical Pharmacies Group Inc.) that provides complete pharmacy care (medication packaging and distribution, clinical support and consulting services) to over 35,000 residents in LTC homes across Ontario, Canada. This was the final phase of a larger project on improving prescribing in LTC. A previous focus group conducted with LTC family physicians identified that warfarin prescribing was one of their key areas of concern. Previous activities included drug utilization reviews, an audit of warfarin prescribing and monitoring practices in our practice area [19], multi-disciplinary education and training (physicians, nursing, and pharmacy), and academic detailing by senior consultant pharmacists and a geriatrician.

### Description of LTC Homes and Study Cohort

Six LTC homes in the province of Ontario, Canada were chosen to pilot this program. The total residents in all six facilities was 1268 (Centre 1: n = 370; Centre 2: n = 184; Centre 3: n = 64; Centre 4: n = 128, Centre 5: n = 234, Centre 6: n = 288). All residents taking warfarin were included in the study cohort, with the exception of residents who had a prosthetic valve. As this was a feasibility study to determine how the system would function in practice, the majority of homes chosen to participate have been involved in past quality improvement studies, or are centres for university residency trainees in primary care. Many have physicians affiliated with an academic health science centre and have access to specialists and academics on a routine basis. Overall, there were 19 prescribing physicians with the number per facility ranging from 1 - 6 physicians.

### Description of MEDeINR Decision Support System

**Figure 1:**
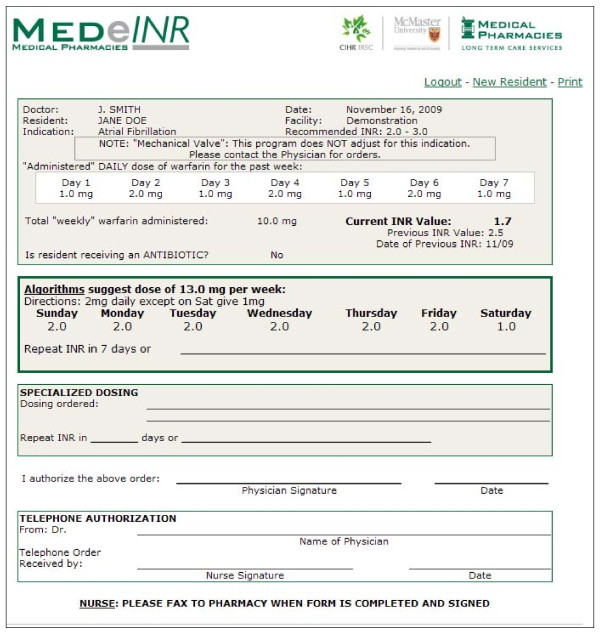
The MEDeINR computer tool

MEDeINR (Figure 1) was designed as a web-based tool to assist LTC staff and clinicians with the day to day management of warfarin. It was available on-line, 24 hours a day. LTC staff, clinicians, and pharmacists were consulted in the development phase as to specific needs and potential barriers and facilitators.

The first step of developing MEDeINR was to modify an algorithm for warfarin dosing validated in acute care/community settings [22] for the elderly patient. Previous studies indicate that warfarin dose requirements decrease with older age [23]. The modified algorithm was developed by an expert in anticoagulation, a geriatrician, and academic and consultant pharmacists. In the next step, computer programmers developed the algorithm into the MEDeINR computer tool which was extensively validated for accuracy.

The MEDeINR system worked in the following way: An INR value is obtained from the laboratory. The user (nurse/clinician/other staff) then logs on to MEDeINR and enters the following data elements: physician name, resident's name, indication for warfarin, current INR value, daily "administered" dose for the 7 days prior to the INR value, current antibiotic information (if applicable). Then, based on the inputs, MEDeINR recommends an overall weekly dose and a specific daily dose is also provided (in order to reduce chance of human error in translating weekly doses to daily doses). For this study, nursing staff printed the MEDeINR recommendation, contacted the physician (via phone or fax), faxed MEDeINR recommendation forms to the pharmacy and also placed the original on the resident's chart. Ultimately any dosing/testing changes were at the discretion of the physician, and the physician could choose not to use the dose suggested by the MEDeINR algorithm and provide an alternative order that was documented by hand in the 'Specialized Dosing' section. Physicians could request access to the on-line tool if they wished to check MEDeINR recommendations themselves.

### Training and Knowledge Dissemination

Selected nurses in the participating LTC homes were chosen to carry out all warfarin monitoring and MEDeINR activities. These nurses were provided with a "user's manual" and in-person training by consultant pharmacists. Physicians were consented and also received training.

### Data Collection and Evaluation

Prior to beginning the study, approval was obtained from the family physicians, Medical Director, Administrator, and Director of Care at each LTC home. Ethics approval was received from the Research Ethics Board at McMaster University. A pre-post implementation study design was utilized to evaluate the MEDeINR system. The study cohort (all patients taking warfarin and without a prosthetic valve) was identified via the central pharmacy database just prior to implementing the MEDeINR system (i.e., considered baseline). Three-months of pre-implementation data was collected via a retrospective chart audit by consultant pharmacists and three-months of prospective data was collected via the MEDeINR computer system (i.e., post-implementation data). All INR data collected via the computer system was also audited/validated by consultant pharmacists via a comparison with laboratory records. Data collected in pre and post-phases included indication for warfarin, recommended INR range, weekly warfarin doses, INR values, and antibiotic information. Further information including demographics, co-morbidities and concomitant drug information were obtained from the central pharmacy database. All residents must have been on warfarin in both the pre and post data collection phases, however some patients may have had fewer than 3-months of INR data in either phase (e.g., if a resident had not been residing in the facility for the entire 3-months during the pre-implementation period or died during the post-implementation phase).

To elicit feed-back about their experience with MEDeINR, a brief on-line survey was sent to LTC staff directly involved in the pilot project. The online survey program *SurveyMonkey *[24] was used to design, collect and store survey responses. The survey was distributed to 15 LTC staff who were directly involved in the MEDeINR pilot within each home. The roles and numbers varied by home, but generally 2-3 people were responsible for coordinating MEDeINR (in most cases it was nurses, but other relevant staff including the Director of care, consultant pharmacist were also surveyed in certain homes). The survey was also sent to 6 physicians. The survey contained 8 closed-ended questions, and 2 open-ended questions where perceptions and beliefs about the system could be expanded upon. Participants were asked about their confidence in using MEDeINR to monitor warfarin and to rate the "user-friendliness" of the computer tool. They were also surveyed about the perceived impact of MEDeINR on INR testing, work-load, team-work, and communication between LTC staff and physicians.

A focus group with three physicians, two nurses, and one consultant pharmacist from two facilities was also conducted to obtain further feed-back. There were two main open-ended questions at the focus group: 1) what aspects of MEDeINR could be improved or changed? 2) what did you like about MEDeINR? A researcher familiar with qualitative methodology led the focus group and results were transcribed and summarized. Several of the co-investigators were present at the focus group so they could address questions and concerns and utilize the knowledge to improve upon the MEDeINR program.

### Statistical Analysis

We compared study outcomes between the pre and post implementation periods using a 'within patient' comparison/paired analysis. All INR values for every resident on warfarin were included, with the exception of residents who had fewer than 2 INR values in either the pre or post phase. Residents who died during the study period were included as long as they had the minimum number of INR values. Rosendaal's [25] method of linear interpolation of INR was used to characterize each day of warfarin therapy: INR values were assigned to each day between INR measurements based on a postulated linear change. Values were rounded to one decimal place. All analyses were conducted overall and for each individual LTC home. Paired t-tests (2-tailed) were utilized to compare the percent of time spent in therapeutic, sub-therapeutic and supratherapeutic range for the pre versus post-implementation periods. Therapeutic range was considered an INR value between 2.0 and 3.0. In secondary analyses, a paired t-test was used to examine pre/post differences in the mean number of INR measures/30 days per resident.

## Results

Our final cohort was 128 residents taking warfarin in six LTC homes, representing 10% of all residents. The mean age of residents taking warfarin was 85.9 [standard deviation (SD) 8.0]; 25 percent were male. The primary indications for taking warfarin were: atrial fibrillation (74%), deep venous thrombosis (20%), pulmonary embolism (6%). Residents had several common co-morbidities (Table 1).

**Table 1:** Baseline patient characteristics, n = 128

Characteristic	No. (percent) or Mean (SD)
Age	85.9 (8.0)
Male	32 (25)
Primary Indication for Warfarin	
Atrial Fibrillation	95 (74)
Deep Venous Thrombosis	16 (20)
Pulmonary Embolism	7 (6)
Co-morbidities	
Hypertension	61 (48)
Coronary Artery Disease	45 (35)
Cerebrovascular Accident	45 (35)
Congestive Heart Disease	21 (16)
Diabetes	21 (16)
COPD	14 (11)
Cardiac Arrythmias	12 (9)
Malignancy	12 (9)
Peripheral Vascular Disease	8 (6)
Other Heart Condition (Murmur, Valvular, Congenital)	6 (5)
Amiodarone use	7 (6)

No. = Number; SD = standard deviation

### INR

**Figure 2:**
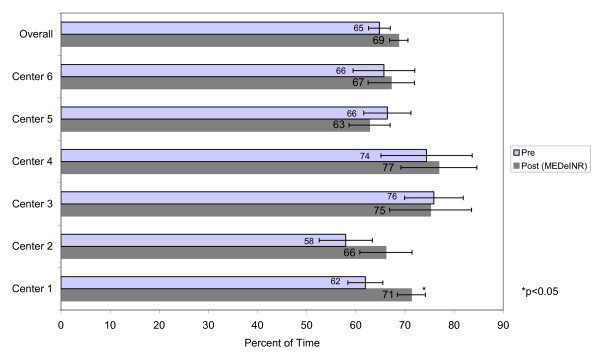
Percent of time spent in therapeutic range

**Figure 3:**
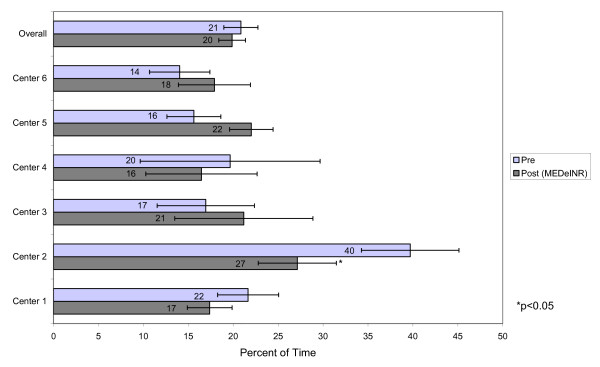
Percent of time spent below therapeutic range

**Figure 4:**
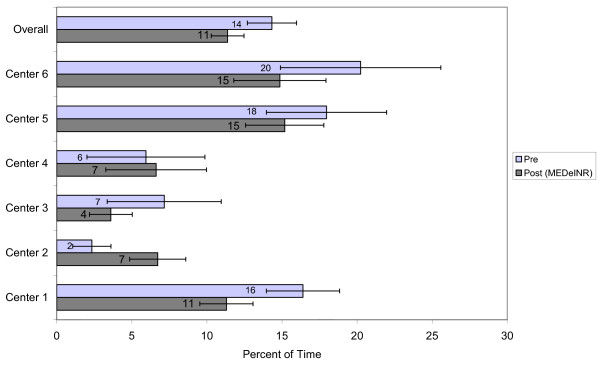
Percent of time spent above therapeutic range

A total of 1308 INR values were recorded during the pre-data phase over 9495 resident days. A total of 1213 INR values were recorded over 11557 resident days during the MEDeINR phase. The mean number of days of INR monitoring per patient (difference between first and last INR dates) was 74 days (range 17-101) in the pre-phase and 90 days (range 27-131) in the post-phase. Figures 2 through 4 compare the percentage of time spent in a) therapeutic range b) below range (subtherapeutic) and c) above range (supratherapeutic) for the pre and post implementation periods. Overall, the TTR increased during the MEDeINR phase (65 to 69%, p = 0.14), but was only significantly increased for Centre 1. There was little change in the pre versus post periods for the subtherapeutic range, with the exception of Centre 2 (Figure 3). The percentage of time spent in supratherapeutic range also decreased from 14% to 11% (p = 0.08) overall, and for Centres 1,3,5 and 6 (Figure 4) but was not significant.

### Testing Frequency

**Figure 5:**
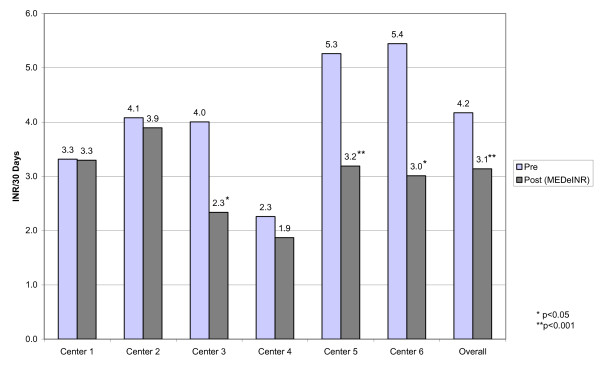
INR testing frequency

Figure 5 displays the INR testing frequency for all residents in our study (overall and by home) between the pre and post implementation periods. The average number of INR tests/30 days per resident was lower in the MEDeINR period overall (4.2 to 3.1, p < 0.001) and for Centre 3 (4.0 to 2.3, p < 0.05), Centre 5 (5.3 to 3.2, p < 0.001) and Centre 6 (5.4 to 3.0, p < 0.001).

### Adverse Events

No major adverse events requiring hospitalization were reported; minor or those not requiring hospitalization were not recorded.

### Survey and Focus Group

Twelve participants (4 physicians, 8 nursing/administration/pharmacy staff) responded to the survey. After learning how to use MEDeINR, 100% of respondents reported that MEDeINR was easy/very easy to use. Compared to previous warfarin monitoring, 75% believed it decreased workload, 17% perceived no change in workload, and 1 respondent (8%) perceived an increase in workload. Compared to previous warfarin monitoring, 80% reported increased confidence using MEDeINR; 20% reported the same level of confidence. When respondents were asked about communication between LTC staff and physicians regarding warfarin management, 92% of respondents felt communication was better (more timely, etc.) with MEDeINR; 8% reported no change. With regards to team-work related to warfarin management, 67% reported that MEDeINR improved team-work and 33% reported no change from the previous system. For respondents from facilities that implemented an INR monitoring/tracking form (n = 9), 67% believed it improved/streamlined INR collection and 33% reported no change (similar to old system). Respondents were also asked about their perceptions of MEDeINR testing frequency: 56% reported a decrease in INR testing, 33% felt it increased INR testing, and 11% reported no change. In focus group feedback, the overall consensus was that the program decreased the work-load, improved confidence in management and decisions, and was generally easy to use. The physicians felt there was a decrease in the phone calls/contacting by staff and fewer INR problems to deal with. Physicians also believed that it decreased some of the anxiety regarding warfarin by nursing staff. Participants believed that more training was needed due to the higher turnover in staffing and to ensure that on-call physicians are aware of the program. To introduce new staff to the program, it was suggested that a video be made for training purposes and that 1 or 2 nursing staff be designated to lead this training with new staff. It was also re-iterated that attaching a tracking sheet with a patient's INR history is an important part of the process (in some facilities this was already implemented).

## Discussion

In this feasibility study, we applied evidence into practice by developing and piloting an electronic decision support system (MEDeINR) for managing warfarin in the LTC environment. To our knowledge, this electronic warfarin system is the first of its kind employed in LTC homes. Warfarin monitoring is an area of concern for may LTC clinicians and staff, and we were able to successfully implement the MEDeINR program into daily practice in our six pilot homes.

When we examined the percentage of time in therapeutic, subtherapeutic and supratherapeutic range in the pre versus post implementation periods, the overall TTR (all homes combined) after implementing MEDeINR increased non-significantly from 65% to 69%, the percentage of time in supratherapeutic range decreased non-significantly from 14% to 11%, and there was little change for the subtherapeutic range.

For this feasibility study, LTC homes were selected as they had either pharmacists or physicians who were involved in quality improvement initiatives and may be "early adopters" of evidence [26,27]. Thus, it is not surprising that before we implemented MEDeINR, the homes in our study had baseline TTR's much higher than previous studies in LTC which report TTR's of 37-51% [2,10,13,16]. All but one home in our study had a baseline TTR above 60% (considered moderately well-controlled [5]). The baseline TTR's were also higher than a previous audit we conducted in our own practice setting, which found an overall TTR in five LTC homes of 54% [19]. Since developing MEDeINR was the final phase of a larger initiative on improving drug prescribing in LTC, some of our pilot homes may have directly or indirectly been influenced by our previous activities (e.g. in-services, academic detailing, and in some cases audit and feedback on prescribing practices which may have included warfarin). Thus, although we did not see a significant increase, our pilot homes either improved or maintained good TTR's. We hypothesize that homes with poorer warfarin control would see greater gains with MEDeINR.

One of our other objectives was to examine how MEDeINR impacted on the frequency of INR testing. An important finding was that the number of INR tests ordered during the MEDeINR period was considerably reduced while at the same time either maintaining or improving TTR. In the homes with the greatest testing reduction (Centres 5 and 6), the number of INR's per 30 days decreased from approximately 5 to 3 in pre versus post-implementation periods. This has implications in terms of quality of life for elderly LTC residents (fewer venipunctures), a decrease in staff time for both laboratory and LTC workers, and a decrease in costs associated with testing.

Given that this was a feasibility study to determine whether MEDeINR could be successfully integrated into daily practice, we obtained feedback from the LTC teams regarding their experiences with MEDeINR. The results of our survey indicate that the majority of respondents believed MEDeINR was easy to use, decreased workload, increased confidence in monitoring warfarin, improved communication between LTC staff and physicians, and improved team-work related to warfarin management. Furthermore, the physicians in our focus group felt there was a decrease in the phone calls/contacting by staff, fewer INR problems to deal with, and a decrease in the anxiety regarding warfarin monitoring by nursing staff. Since LTC physicians must rely in part on the LTC team to provide appropriate warfarin monitoring, the benefit of MEDeINR is that it provided support for the entire LTC team. Interestingly, a prior survey of LTC physicians indicated that although the majority agreed an anticoagulation service would be beneficial for improving TTR's and reducing workload, approximately half believed an anticoagulation service would intrude on physician decision-making and they would not utilize one for LTC residents [28]. From our feedback, it appeared MEDeINR was a good balance between decision making and convenience that LTC physicians may desire.

One of our primary limitations was that we may not have had adequate sample size, particularly in the smaller homes, to see potentially significant changes in TTR and INR testing frequency. Another limitation was the lack of a run-in period after implementation of MEDeINR (to work out any initial bugs), so it is possible our estimates of TTR during the MEDeINR phase are underestimated.

Implementation of the program was not without its challenges. As issues arise, support and infrastructure is needed to deal with questions and concerns and ongoing education is needed to ensure proper use and when staff turn-over occurs. This process was led by the pharmacists with support from the investigative team. Although the overall response to the program was positive, in one home, it was noted by the Director of Care that the physicians may not have been fully aware of the program and as a result were not interested in its continued use. One of the suggestions in feedback received from staff was that a video be made for training purposes. Confusing terms, implementation and process issues have been discussed and improved upon in keeping with the idea that translating knowledge to action is an "iterative, dynamic and complex process" [20].

Despite some of the challenges and barriers to warfarin management in the LTC environment, there are several facilitators that actually make the LTC environment an ideal setting to manage warfarin (versus patients in the community or acute settings). For example, the supervised medication administration and the availability of routine laboratory draws on-site are both added benefits. Since an anticoagulation service may not be feasible in the LTC setting, MEDeINR builds on the team-based approach to care and the closely supervised environment of LTC homes. Although we did not formally examine the impacts on work-load and costs, the perception was that MEDeINR had a beneficial effect on these and we know quantitatively that residents had fewer INR-related venipunctures.

The uptake of MEDeINR system in our pilot nursing homes demonstrates that it was a practical, usable clinical information system that can be incorporated into the LTC environment. The final step of the "knowledge to action" framework is ensuring sustained use of knowledge [20,21]. At every step, the end-users of the knowledge - the LTC teams - were included to ensure that MEDeINR met their needs and necessary changes were made to improve upon the system. Since this was a cooperative approach, integrating both academia and industry, we believe that MEDeINR can be sustained. Our partner pharmacy provider has already implemented MEDeINR in 30 homes. Our next steps will be to evaluate MEDeINR at a wider level, including homes that have not been influenced by other prescribing initiatives and over a longer time period. With a larger sample size and duration, we will also examine whether MEDeINR has any effect on reducing serious hemorrhagic and thromboembolic events.

## Competing interests

The authors declare that they have no competing interests.

## Authors' contributions

AP and CCK and contributed to conception and design, data acquisition, analysis and interpretation of data, and drafting of the manuscript; GC contributed to conception and design, data acquisition, testing and verification of the MEDeINR application, analysis and interpretation of data and critical review of the manuscript; JBS contributed to conception and design, testing and verification of the application, data acquisition, analysis and interpretation of the data, and critical review of the manuscript. In addition, JBS coordinated the development of the computer application and provided specifications for the program developers. LW, LD, and MAC contributed to conception and design, analysis and interpretation of the data and critical review of the manuscript. All authors have given final approval of the version to be published.

## Authors' information

AP is a geriatrician at Hamilton Health Sciences, a Professor of Medicine at McMaster University, Hamilton, Ontario, Canada, and the Canadian Institutes of Health Research and Eli Lilly Canada Chair in Osteoporosis. CCK and LW are Research Associates at McMaster University. GC is a clinical consultant pharmacist and JS is a pharmacist and Manager, Systems Services for Medical Pharmacies Group Inc., Ontario, Canada. LD is an academic pharmacist and Associate Professor of Family Medicine and Clinical Epidemiology and Biostatistics at McMaster University. MAC is Chair, Division of Hematology & Thromboembolism and Professor of Medicine & Pathology and Molecular Medicine at McMaster University. MAC is also a Career Investigator, Heart and Stroke Foundation of Ontario.

## Pre-publication history

The pre-publication history for this paper can be accessed here:

http://www.biomedcentral.com/1471-2318/10/38/prepub
